# Polyacrylic Acid-Ca(Eu) Nanoclusters as a Luminescence Sensor of Phosphate Ion

**DOI:** 10.3390/nano12142398

**Published:** 2022-07-14

**Authors:** Chunhui Song, Qifa Song, Ziyou Ding, Yingchao Han

**Affiliations:** 1 State Key Laboratory of Advanced Technology for Material Synthesis and Processing, Wuhan University of Technology, Wuhan 430070, China; sch1997@whut.edu.cn (C.S.); songqifa@whut.edu.cn (Q.S.); dingziyou@hot.mail.com (Z.D.); 2 Foshan Xianhu Laboratory of the Advanced Energy Science and Technology Guangdong Laboratory, Xianhu Hydrogen Valley, Foshan 528200, China

**Keywords:** nanoclusters, Eu^3+^ luminescence sensor, PO_4_^3−^ detection, charge transfer band

## Abstract

In this study, we synthesized polyacrylic acid (PAA)-Ca (Eu) nanoclusters as a luminescence sensor of phosphate ion by a complex method, and we aimed to achieve the quantitative detection of PO_4_^3−^ based on the sensitivity of the charge transfer band of Eu^3+^ to anionic ligand. The resulting PAA-Ca(Eu) nanoclusters showed a well-dispersed and a dot-like morphology, with an ultra-small diameter (the average size of 2.17 nm) under high resolution transmission electron microscopy(HRTEM) observation. A dynamic light scattering particle size analyzer (DLS) showed a hydrodynamic size of 2.39 nm. The (PAA)-Ca (Eu) nanoclusters as a luminescence sensor showed a significantly higher sensitivity for PO_4_^3−^ than other anions (CO_3_^2−^, SiO_3_^2−^, SO_4_^2−^, SO_3_^2−^, Br^−^, Cl^−^, F^−^). The luminescence intensity displayed a linear increase (y = 19.32x + 74.75, R^2^ > 0.999) in a PO_4_^3^ concentration range (0–10 mM) with the concentration of PO_4_^3−^ increase, and the limit of detection was 0.023 mM. The results showed good recovery rates and low relative standard deviations. These (PAA)-Ca (Eu) nanoclusters are hopeful to become a luminescence sensor for quantitatively detecting PO_4_^3−^.

## 1. Introduction

Europium element with a unique 4f electron layer structure is a commonly used luminescent probe [1,2,3] due to its good optical stability, high thermal and chemical stability, narrow emission band, high resistance to photobleaching, and light quenching [4,5,6]. The excitation wavelength of Europium mainly includes the 350–475 nm band of energy levels transition and the charge transfer band (CTB) in the ultraviolet region [5,7]. The energy level transition excitation can obtain better near-infrared emission luminescence, which is mainly used in the biomedical field [6,8,9,10,11,12]. The CTB has unique properties, Eu^3+^ binds to the anionic ligand to form a CTB. The position of the charge transfer transition band depends on the ligand [13,14,15,16,17,18]. Therefore, the CTB of Eu^3+^ can be used for qualitative and quantitative analysis of the types and contents of anionic ligands. For example, CTB formed with phosphate in hydroxyapatite is at 254 nm, while CTB formed with anionic ligand in LaOF is at 285 nm [7,19].

Phosphorus plays an important role in organisms and the environment [20,21]. Excessive phosphate content in water can cause water pollution [22,23]. Phosphate in organisms participates in a variety of metabolism processes. Phosphate content is one of the important indicators of human health, and its quantitative detection is of great significance [24,25]. In this study, inspired by the biomineralization process of calcium phosphate, we used polyacrylic acid (PAA) to complex Ca^2+^ and Eu^3+^ ions to obtain PAA-Ca (Eu) nanoclusters as a sensor for the quantitative detection of PO_4_^3^^−^ based on the sensitivity of charge transfer band of Eu^3+^ to anionic ligand. The morphology, size, ion selectivity and luminescence of PAA-Ca (Eu) nanoclusters were characterized, and the mechanism of quantitative phosphate radical detection was analyzed and explained by luminescence spectra and molecular dynamics simulation (MDS).

## 2. Materials and Methods

### 2.1. Synthesis of PAA-Ca (Eu) Nanoclusters

The PAA-Ca(Eu) nanoclusters were prepared by a complex method. An aqueous Ca(Eu) solution (20 mL) was prepared using CaCl_2_·2H_2_O (99.42 mg, Sinopharm, Beijing, China) and Eu(NO_3_)·6H_2_O (33.52 mg, Aladdin, Shanghai, China) with a concentration of 37.575 mM in which the Eu^3+^/(Ca^2+^ + Eu^3+^) molar ratio was 10%. The solution was stirred vigorously to make it fully dissolved. An aqueous solution of PAA (average molecular weight of ~1800 g/mol, 216.43 mg, 20 mL, Sigma, St. Louis, USA) was quickly added to the aqueous Ca(Eu) solution, and the pH was adjusted to 7.5–8.0 using NH_3_·H_2_O (Sinopharm, Beijing, China) to yield the PAA-Ca(Eu) nanoclusters. The temperature of all the above solutions was room temperature (25 °C).

### 2.2. Characterization

High resolution transmission electron microscopy (HRTEM, Talos F200S, Waltham, MA, USA) was used to observe and to analyze the microstructure of the materials. Fourier transform infrared spectroscopy (FT-IR, Nicolet6700, Waltham, MA, USA) was used to record the spectra of the near infrared region (4000~400 cm^−1^), analyze and study the vibration mode of the characteristic peak of the material, identify the substance, and determine the chemical composition or relative content of the substance. A dynamic light scattering particle size analyzer (DLS, Malvern, UK) was used to measure the particle size distribution and the dispersion coefficient of solution. Luminescence excitation and emission spectra of samples were measured by luminescence spectrophotometer (970CRT, Shanghai Sanco, Shanghai, China).

### 2.3. Detection of PO_4_^3−^

An aqueous solution of phosphate ion was prepared by Na_2_HPO_4_·12H_2_O and added to the PAA-Ca(Eu) nanoclusters solution. Finally, NH_3_·H_2_O was used to adjust the pH to 9.0–9.5 for luminescence detection.

### 2.4. Preparation of Buffer Solution

A total of 1.07 g of NH_4_Cl (Sinopharm, Beijing, China) was added to 100 mL of deionized water. After it was fully dissolved, ammonia was added to adjust the pH of the aqueous solution to 8.0 to obtain the buffer solution.

### 2.5. Molecular Dynamics Simulation

All MDS employed the AMBER/general AMBER force field. In the cubic simulation unit with an initial size of 10 nm, the step change was set to 1 fs, and all simulations were run for 50 ns in real time using Gromacs 2018 software package [26,27].

## 3. Results and Discussion

### 3.1. Structure Characterization

**Figure 1:**
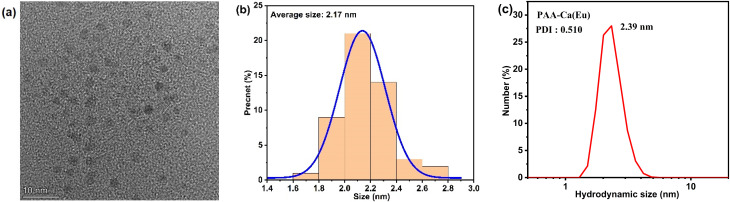
(**a**) High-resolution transmission electron microscopy image of PAA-Ca (Eu) nanoclusters; (**b**) Particle size statistics of (**a**); (**c**) Hydrodynamic size of PAA-Ca(Eu) nanoclusters.

First, the microstructure and the particle size of PAA-Ca (Eu) nanoclusters were characterized (Figure 1). Through HRTEM, it can be seen that the nanoclusters present dot-like particles, and the nanoclusters do not gather directly. The particle size also presents a relatively uniform distribution. Through the statistics of the nanoclusters in the HRTEM image, their particle size is concentrated in the range of 1.8–2.4 nm (this particle size range accounts for 88% of the total particle size), with an average particle size of 2.17 nm. DLS test results also showed a similar hydrodynamic size (2.39 nm).

**Figure 2:**
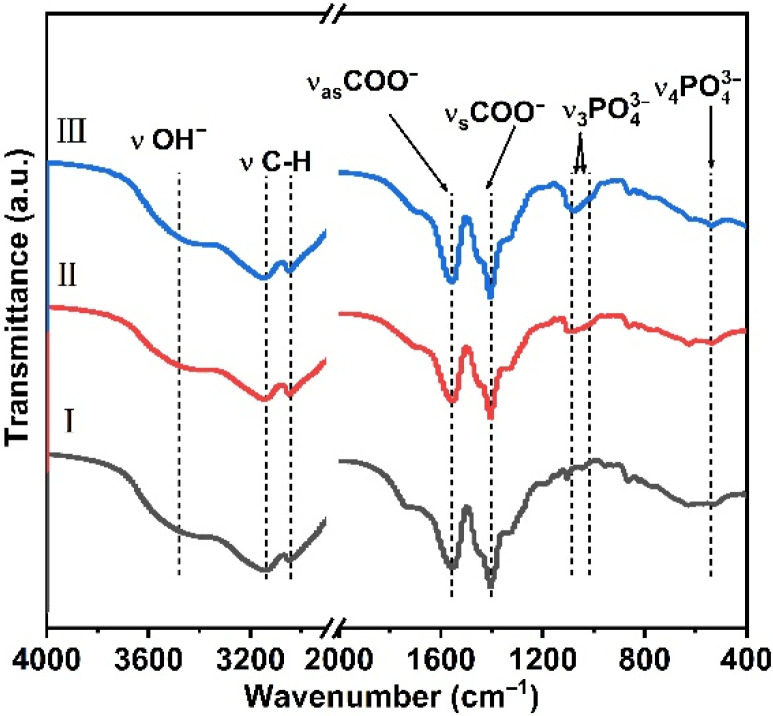
Fourier transform infrared spectroscopy spectra of PAA-Ca (Eu) nanoclusters with different PO_4_^3−^ concentration. Ⅰ–Ⅲ are 0 mM, 2 mM, and 7.5 mM.

In addition, FT-IR spectra of PAA-Ca (Eu) nanoclusters and samples with different PO_4_^3−^ additions are shown in Figure 2. The absorption peak at 3478 cm^−1^ is the O-H stretching vibration peak in PAA molecule [28]. The absorption peaks at 1556 cm^−1^ and 1401 cm^−1^ are the asymmetric stretching vibration peak (ν_as_(COO^−^)) and the symmetric stretching vibration peak (ν_s_(COO^−^)) of COO^−^ in the PAA molecule, respectively. Compared with pure PAA, the C=O absorption peak shifts to a low frequency and the C-O absorption peak shifts to a high frequency, which ν_as_(COO^−^)–ν_s_(COO^−^) is approximately 150 cm^−1^, indicating that the coordination between carboxylic acid and the metal ions in PAA is a bridge coordination compound [29,30]. After adding PO_4_^3−^, the absorption peak of the phosphate ion appeared obviously in the infrared spectrum, which was located at 1104 cm^−1^, 1072 cm^−1^ and 536 cm^−1^, belonging to the asymmetric stretching (ν_3_) and the asymmetric angle change (ν_4_) of PO_4_^3−^ [31,32].

### 3.2. Luminescent Characterization

#### 3.2.1. Ion Selectivity

**Figure 3:**
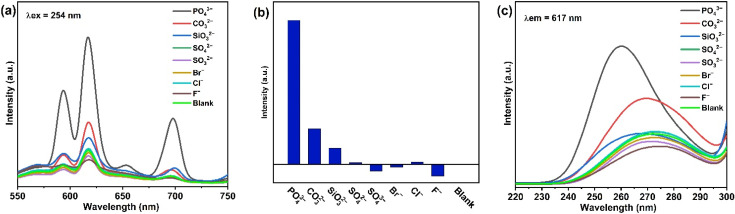
(**a**) Emission spectra (λex = 254 nm) of different anions at the excitation wavelength of 254 nm; (**b**) Luminescence intensity of the characteristic emission peak at 617 nm was selected for comparison; (**c**) Excitation spectra (λem = 617 nm) of different anions at emission wavelengths of 617 nm.

PAA-Ca(Eu) nanoclusters were used as sensors to detect common anions (the anion concentration was 10 mM). As shown in Figure 3a, PO_4_^3−^ is the most sensitive to the sensor, and it has the highest luminescence intensity. The luminescence emission peak with the maximum luminescence intensity (617 nm) was selected for comparison, as shown in Figure 3b. It can be more intuitively observed that the sensor is sensitive to PO_4_^3−^. Figure 3c shows that CTB positions and intensities are different for different anionic ligands. The CTB of PO_4_^3−^ position is unique, and it is the strongest. All of the above indicated that PAA-Ca (Eu) nanoclusters could be used for the detection of PO_4_^3−^ concentration.

#### 3.2.2. Detection of PO_4_^3−^ Concentration

**Figure 4:**
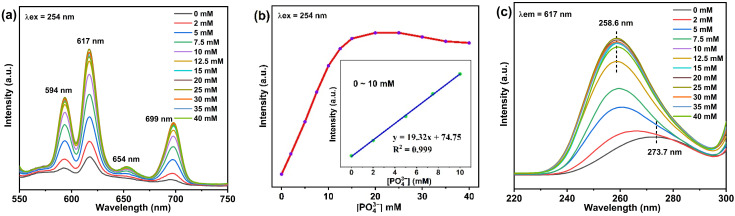
(**a**) Emission spectra (λex = 254 nm) of PAA-Ca(Eu) nanoclusters and PO_4_^3−^ at different concentrations; (**b**) The relationship between luminescence intensity increase rate and PO_4_^3−^ concentration at 617 nm emission peak; (**c**) Excitation spectra (λem = 617 nm) of PAA-Ca(Eu) nanoclusters and PO_4_^3−^ at different concentrations.

In the emission spectrum excited at 254 nm, Eu^3+^ showed characteristic emission at 594 (^5^D_0_ → ^7^F_1_), 617 (^5^D_0_ → ^7^F_2_), 654 (^5^D_0_ → ^7^F_3_), and 699 nm (^5^D_0_ → ^7^F_4_) (Figure 4a). Figure 4b shows that with the increase of PO_4_^3−^ concentration, the increase of luminescence first increased and then remained basically unchanged. The linear fitting of PO_4_^3−^ concentration in the range of 0–10 mM showed that the linear equation was y = 19.32x + 74.75, and its R^2^ was 0.999, indicating that PAA-Ca(Eu) nanoclusters can quantitatively detect PO_4_^3−^ in this concentration range. In the excitation spectrum, Eu-O CTB gradually moved to the left from 273.7 nm to 258.6 nm with the increase of PO_4_^3−^ concentration, indicating that the anion ligand connected to Eu^3+^ changed during this process.

LOD = 3σ/K(1)

The detection limit of the fluorescent sensor is calculated using Formula (1), where LOD is limit of detection, σ is the standard deviation of the blank, and K is the slope of the linear relationship. We tested six groups of blank samples, obtained their standard deviation, and calculated that the detection limit of the luminescence sensor for PO_4_^3−^ was 0.023 mM. It shows that the sensor can be used to detect PO_4_^3−^ in serum and other samples [33]. We added a known concentration of PO_4_^3−^ to the sample, which reacted with PAA-Ca(Eu) nanoclusters, and then tested its luminescence at 254 nm excitation wavelength. According to the emission peak intensity at 617 nm and the linear equation in Figure 4b, the spiked recovery rate of PO_4_^3−^ in the sample was calculated. The results are shown in Table 1. Overall, all samples showed good recovery rates and low relative standard deviations (RSD) within the linear range, making PAA-Ca(Eu) nanoclusters a sensor for PO_4_^3−^ quantitative detection.

#### 3.2.3. Buffer Solution

**Figure 5:**
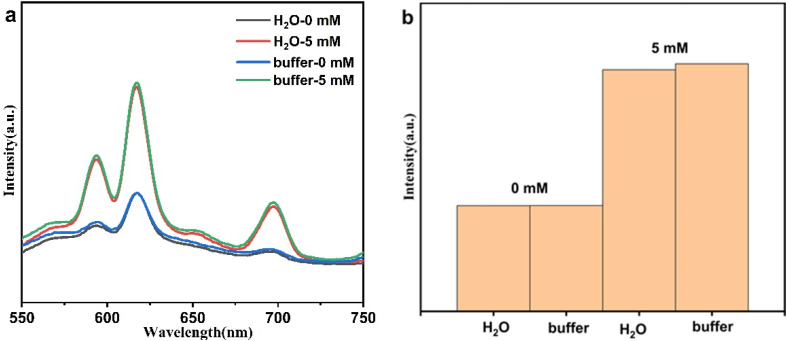
(**a**) The emission spectrum of PAA-Ca(Eu) nanoclusters in aqueous solution and buffer solution after reacting with different concentrations of PO_4_^3−^, (**b**) luminescence intensity at 617 nm.

It can be seen from Figure 5 that in an aqueous solution and a buffer solution, the luminescence intensity of the PAA-Ca(Eu) nanoclusters is basically the same after reacting with PO_4_^3−^ of the same concentration. It proved that the luminescence sensor also has a good sensing function in the buffer solution.

### 3.3. Mechanism of PO_4_^3−^ Concentration Detection

**Figure 6:**
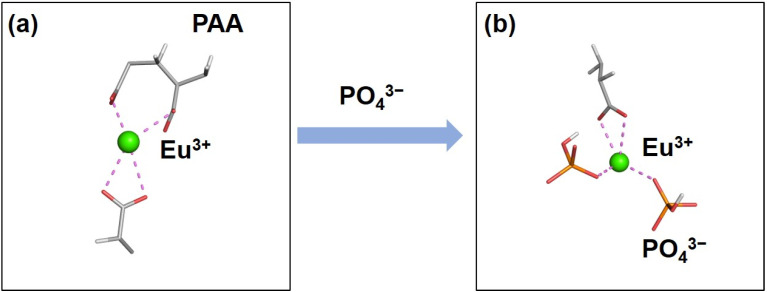
(**a**) The bonding of Eu in PAA-Ca(Eu) nanoclusters; (**b**) The bonding of Eu after adding PO_4_^3−^.

After adding PO_4_^3−^ to PAA-Ca(Eu) nanoclusters, the vibrational peak of PO_4_^3−^ appeared in FT-IR, and the peak position and intensity of CTB changed in the excitation spectra (λem = 617 nm), indicating that the anions bonded with Eu changed in this process. In addition, MDS showed that Eu^3+^ combines with the oxygen anion of the PAA carboxyl group in PAA-Ca(Eu) nanoclusters, showing Eu–O_1_ CTB (Figure 6a). When PO_4_^3−^ was added to the PAA-Ca(Eu) nanoclusters, the COO^–^ bonded Eu^3+^ was bound by the oxygen anion of PO_4_^3−^, displaying a new Eu–O_2_ CTB (Figure 6b). This change in the bonding state of Eu^3+^ caused an increased energy state, corresponding to the shift to a low wavelength and an increased luminescence intensity. Based on this mechanism, the quantitative detection of PO_4_^3−^ can be realized.

## 4. Conclusions

In conclusion, we synthesized ultra-small PAA-Ca(Eu) nanoclusters with an average particle size of 2.17 nm under HRTEM observation. The nanoclusters are sensitive to PO_4_^3−^, and they can be used for quantitative detection of PO_4_^3−^ in a certain concentration range (0–10 mM), with good linear correlation. The LOD is 0.023 mM. Based on the sensitivity of CTB of Eu^3+^ to anionic ligand, the quantitative detection of PO_4_^3−^ can be carried out. In addition, the detected concentration range by the PAA-Ca(Eu) nanoclusters sensor covers the content of PO_4_^3−^ in serum, urine, and sewage. So, it is hoped that it can detect PO_4_^3−^ in physiological conditions and a natural environment.

## Figures and Tables

**Table 1:** Results and recovery of samples (*n* = 3).

PO_4_^3^^−^ Spiked (mM)	PO_4_^3^^−^ Found (mM)	Recovery (%)	RSD (%)
1	1.060	106.0	4.2
4	4.200	105.0	
5	4.793	95.9	
8	7.951	99.4	
10	9.914	99.1	

## Data Availability

All data supporting the research results are provided in this paper, and relevant data are also available from corresponding authors. The original data for relevant Figures can also be obtained from corresponding authors.
